# Phenotypic profile of expanded NK cells in chronic lymphoproliferative disorders: a surrogate marker for NK-cell clonality

**DOI:** 10.18632/oncotarget.5480

**Published:** 2015-11-06

**Authors:** Paloma Bárcena, María Jara-Acevedo, María Dolores Tabernero, Antonio López, María Luz Sánchez, Andrés C. García-Montero, Noemí Muñoz-García, María Belén Vidriales, Artur Paiva, Quentin Lecrevisse, Margarida Lima, Anton W. Langerak, Sebastian Böttcher, Jacques J.M. van Dongen, Alberto Orfao, Julia Almeida

**Affiliations:** ^1^ Cancer Research Centre (IBMCC, CSIC-USAL), Institute of Biomedical Research of Salamanca (IBSAL), (NUCLEUS) and Department of Medicine, University of Salamanca, Salamanca, Spain; ^2^ Research Unity and IECSCYL, University Hospital of Salamanca and IBSAL, Salamanca, Spain; ^3^ Department of Hematology and Institute of Biomedical Research of Salamanca (IBSAL), University Hospital of Salamanca, Salamanca, Spain; ^4^ Unidade de Gestão Operacional em Citometria, Serviço de Patologia Clínica, Centro Hospitalar e Universitário de Coimbra, Instituto Politécnico de Coimbra, ESTESC-Coimbra Health School, Análises Clínicas e Saúde Pública, Coimbra, Portugal; ^5^ Department of Hematology, Laboratory of Cytometry, Hospital de Santo António, Centro Hospitalar do Porto, and Unit for Multidisciplinary Research in Biomedicine (UMIB), Porto, Portugal; ^6^ Department of Immunology, Erasmus MC, University Medical Center, Rotterdam, The Netherlands; ^7^ Medical Clinic II, University Medical Center Schleswig-Holstein, Campus Kiel, Kiel, Germany

**Keywords:** natural killer cells, NK cells, immunophenotype, clonality, CLPD-NK

## Abstract

Currently, the lack of a universal and specific marker of clonality hampers the diagnosis and classification of chronic expansions of natural killer (NK) cells. Here we investigated the utility of flow cytometric detection of aberrant/altered NK-cell phenotypes as a surrogate marker for clonality, in the diagnostic work-up of chronic lymphoproliferative disorders of NK cells (CLPD-NK). For this purpose, a large panel of markers was evaluated by multiparametric flow cytometry on peripheral blood (PB) CD56^low^ NK cells from 60 patients, including 23 subjects with predefined clonal (*n* = 9) and polyclonal (*n* = 14) CD56^low^ NK-cell expansions, and 37 with CLPD-NK of undetermined clonality; also, PB samples from 10 healthy adults were included. Clonality was established using the human androgen receptor (HUMARA) assay. Clonal NK cells were found to show decreased expression of CD7, CD11b and CD38, and higher CD2, CD94 and HLADR levels *vs*. normal NK cells, together with a restricted repertoire of expression of the CD158a, CD158b and CD161 killer-associated receptors. In turn, NK cells from both clonal and polyclonal CLPD-NK showed similar/overlapping phenotypic profiles, except for high and more homogeneous expression of CD94 and HLADR, which was restricted to clonal CLPD-NK. We conclude that the CD94^hi^/HLADR^+^ phenotypic profile proved to be a useful surrogate marker for NK-cell clonality.

## INTRODUCTION

Chronic lymphoproliferative disorders (CLPD) of natural killer (NK) cells (CLPD-NK) are a relatively rare and heterogeneous group of diseases characterized by a persistent (> 6 months) increase of mature-appearing NK cells (> 2 × 10^9^/L) in peripheral blood (PB), in the absence of a clearly identifiable cause [1, 2, 3]. Expanded NK cells usually display a large granular lymphocyte (LGL) morphology and a typical surface-membrane CD3^−^, CD2^+^, CD16^+^, CD56^low^ and CD57^−/+^ immunophenotype [4, 5]. From the clinical point of view, most patients present with an indolent course in the absence of any symptoms [1, 6, 7, 8]; without treatment, the number of circulating PB NK cells usually remains stable for long periods of time, and some cases have even been reported to show spontaneous regression [9, 10]. Actually, CLPD-NK have recently emerged as a new (provisional) entity in the WHO2008 (World Health Organization 2008) classification of lymphoid malignancies [11], mostly because of the need for distinguishing such CLPD-NK cases from more aggressive NK-cell leukemias/lymphomas [12].

The WHO2008 category of CLPD-NK comprises a broad spectrum of NK-cell expansions, ranging from reactive to monoclonal/neoplastic disorders, which have been previously denominated as *chronic NK-cell lymphocytosis*, *chronic NK-large granular leukemia* or *NK-cell LGL lymphocytosis*, among other terms [7, 13, 14]. Hence, a major challenge for the diagnosis of CLPD-NK remains the ability to assess the reactive (polyclonal/oligoclonal) *vs*. (mono)clonal nature of the expanded NK cells, due to the lack of an universal and specific marker for NK-cell clonality. Therefore, because clonal NK cells from CLPD-NK typically do not show immunoglobulin or T-cell receptor gene rearrangements and their karyotype is normal in most cases [10, 13, 15], alternative methods have been used as surrogate markers for NK-cell clonality –including detection of EBV^+^ NK cells by Southern blotting [16, 17, 18, 19, 20], analysis of restriction fragment length polymorphisms (RLFP) and restricted (or aberrant) expression of a single isoform of KIR receptors [21, 22, 23, 24], the assessment of the pattern of inactivation of the X-chromosome (e.g. the human androgen receptor assay –HUMARA-) [25, 26] and more recently, the presence of *STAT3* mutations [27]; however, these approaches only proved to work in a fraction of patients with chronic NK-cell expansions [18, 19, 20, 23, 24], or they showed inconclusive results [22, 27, 28, 29]. Therefore, since a universal and specific marker for NK-cell clonality is still lacking, precise definition of those immunophenotypic profiles specifically associated with NK-cell clonality, could contribute to the distinction between reactive and clonal CLPD-NK.

In the present study we investigated the immunophenotypic profile of expanded NK cells from 23 females (selected from a total of 60 patients) with predefined monoclonal and polyclonal CLPD of CD56^low^ NK cells *vs*. that of normal PB NK cells, using a large panel of 26 markers analyzed by multiparameter flow cytometry; ultimately, we aimed at identifying aberrant immunophenotypes that could be used as surrogate markers for NK cell clonality. Overall, our results showed that despite multiple immunophenotypic differences existed between normal and expanded CD56^low^ NK cells, the distinction between monoclonal and polyclonal NK cells mostly relied on a strong and more homogeneous pattern of expression of HLADR and CD94, typically restricted to clonal CLPD-NK.

## RESULTS

### Clinical and laboratory characteristics of patients with monoclonal vs. polyclonal expansions of PB CD56^low^ NK-cells

From the 23 female patients who were heterozygous for the HUMARA assay, 9 were classified as having monoclonal and 14 as polyclonal NK-cell expansions. Analysis of somatic mutations of the signal transducer and activator of transcription 3 and 5b genes (*STAT3* and *STAT5b*) were consistent with the HUMARA assay, as the hotspot D661Y (c.1981G > T) mutation at the SH2 (exon 21) domain of *STAT3* was detected in 1/4 cases carrying clonal NK cells, while no mutations were found in any of the polyclonal cases screened for *STAT3* and *STAT5b* genes (*n* = 5).

From the clinical point of view, no statistically significant differences were found between the polyclonal and monoclonal cases. Briefly, both groups showed indolent disease, in the absence of organomegalies, recurrent infections, associated neoplasias and/or autoimmune disorders, except for two monoclonal CD56^low^ NK-cell cases, who presented skin lesions, associated in one of them with immune thrombocytopenia (Table 1).

**Table 1:** Clinical and laboratory characteristics of subjects with monoclonal *vs*. polyclonal expansions of PB CD56^low^ NK cells vs. normal CD56^low^ NK cells

	Healthy adults (*n* = 10)	Cases with expanded/aberrant CD56^low^ NK cells		*P*-value
		Polyclonal cases (*n* = 14)	Monoclonal cases (*n* = 9)	
Age (years)*	47 ± 24 (27–87)	55 ± 16 (42–83)	54 ± 26 (25–81)	NS
**Reason for consulting:**				
Routine blood analysis	NA	83%	80%	NS
General symptoms	NA	17%	20%	NS
**Physical examination:**				
Skin lesions	0%	0%	25%	NS
**Associated autoimmune diseases**	0%	0%	13%**^§^	NS
**Laboratory parameters:**				
Leukocytosis (>10 × 10^9^/L)	0%	17%	25%	NS
Lymphocytosis (>5 × 10^9^/L)	0%	33%	38%	**0.04^a^**
Lymphocytosis (>3 × 10^9^/L)	**0%**	**58%**	**100%**	**≤ 0.05^a^,^b^,^c,d^**
Neutropenia (<1.5 × 10^9^/L)	0%	8%	13%	NS
Anemia (<100g/L)	0%	17%	0%	NS
Thrombocytopenia **(<**100×10^9^/L)	0%	25%	13%^§^	NS
PB cell counts*				
**Hemoglobin** (g/L)	135 ± 17 (160–97)	124 ± 26 (88–186)	139 ± 13 (114–159)	NS
**N. of platelets** (× 10^9^/L)	233 ± 61 (159–327)	240 ± 120 (79–476)	218 ± 86 (83–346)	NS
**WBC count** (× 10^9^/L)	6.1 ± 3.1 (4.5–8.2)	7.1 ± 3.4 (3.9–15.5)	8.7 ± 3.6 (4.2–15.4)	NS
**N. of neutrophils** (× 10^9^/L)	3.6 ± 0.8 (2.4–4.9)	2.6 ± 1.6 (0.9–7.4)	3.2 ± 2.4 (0.3–8.3)	**0.02^a^**
**N. of lymphocytes** (× 10^9^/L)	1.9 ± 0.7 (0.7–2.8)	3.8 ± 1.9 (1.6–7)	4.7 ± 1.3 (3.1–6.6)	**≤ 0.01^a^,^b^,^c^**
**N. of CD56^low^**NK cells**** (× 10^9^/L)	0.25 ± 0.1 (0.1–0.4)	1.2 ± 0.6 (0.6–2.2)	2.2 ± 1.2 (0.6–4)	**≤ 0.001^a^,^b^,^c^**
% of CD56^low^ NK cells*				
From all PB leucocytes	4.2 ± 1.3 (2–6)	20 ± 7 (7–33)	28 ± 12 (14–43)	**≤ 0.001^a^,^b^,^c^**
From PB lymphocytes	**13 ± 13 (8–19)**	**38 ± 10 (20–50)**	**55 ± 11 (38–67)**	**≤ 0.003^a^,^b^,^c^,^d^**

Results expressed as percentage of cases or (*) as mean ± one standard deviation (range).

*P*-values correspond to the following group comparisons:

a healthy adults *vs*. both polyclonal plus monoclonal CD56^low^ NK-cell expansions;

b healthy adults *vs*. polyclonal CD56^low^ NK-cell expansions;

c healthy adults *vs*. monoclonal CD56^low^ NK-cell expansions;

d polyclonal *vs*. monoclonal CD56^low^ NK-cell expansions.

PB: peripheral blood. NA: not applicable. NS: no statistically significant differences found (*p* > 0.05).PB: peripheral blood. WBC: white blood cells.

None of the cases studied showed lymphadenopathies, hepatosplenomegaly and/or associated neoplasias.

§ One case diagnosed with immune thrombocytopenia.

Despite the above referred similarities, all cases with monoclonal CD56^low^ NK cells had lymphocytosis > 3 × 10^9^/L at presentation, while this occurred in only 58% of cases with polyclonal CD56^low^ NK cells (*p* = 0.05). As expected, increased lymphocyte counts in both groups were at the expense of an increased number of PB CD56^low^ NK cells (*p* ≤ 0.001 *vs*. healthy donors), in both absolute and relative numbers (Table 1); of note, the increase in the percentage of NK cells from all PB lymphocytes, was significantly higher among those patients in whom clonality was confirmed *vs*. polyclonal cases (Table 1).

### Immunophenotypic features of monoclonal vs. normal/reactive polyclonal PB CD56^low^ NK cells

**Figure 1:**
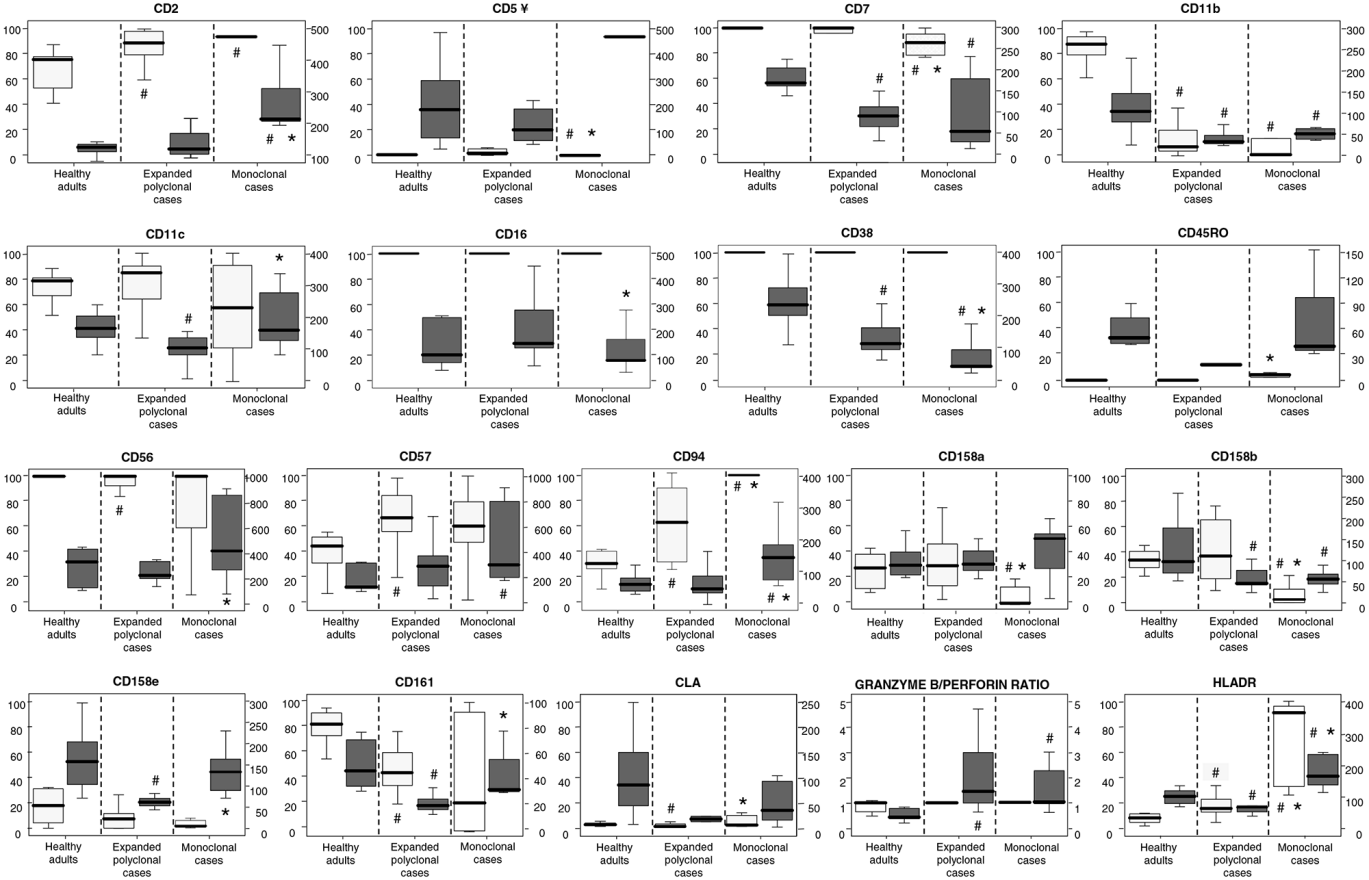
Immunophenotypic characteristics of expanded monoclonal versus expanded polyclonal and normal adult peripheral blood CD56^low^ NK cells Clonality was assessed in all patients included in the analyses displayed (*n* = 23) by the HUMARA assay. CD56^low^ NK cells from the three groups of subjects showed a similar positive reactivity for CD8, CD45RA and CD45, while the expression of CD3, CD25, CD27, CD28 and CD197 (CCR7) was systematically negative. Results are expressed for each marker as percentage of positive cells (light gray boxes, referred to the scales at the left of the plots) and mean fluorescence intensity - MFI- of positive cells - arbitrary relative linear units scaled from 0 to 10^4^ cells (dark gray boxes, referred to the scales at the right of the plots). Notches boxes represent 25th and 75th percentile values; the line in the middle and vertical lines correspond to the median value and the minimum-maximum values (without the extreme values and outliers), respectively. **^¥^** Please note that the high expression levels of CD5 for clonal cases corresponds only to one case in which 1% of CD56^low^ NK cells were CD5^+^ with a MFI of 482. # *P*-value < 0.05 *vs*. CD56^low^ NK cells from the control group (healthy adults).*P-value < 0.05 *vs*. expanded polyclonal CD56^low^ NK cells.

Overall, expanded CD56^low^ NK cells from both monoclonal (*n* = 9) and polyclonal (*n* = 14) cases showed a similar immunophenotypic profile, which was clearly different from that of normal PB CD56^low^ NK cells from healthy controls: increased levels of CD2, CD57, CD94, HLADR, granzyme B and perforin, and lower expression of CD7, CD11b, CD38 and the CD161 and CD158b killer receptors (*p* < 0.05) (Figure 1 and Supplementary Figure S1). Despite this, expanded CD56^low^ NK cells from monoclonal cases showed significantly greater levels of both CD94 and HLADR, with also increased percentages of both CD94^+^ and HLADR^+^ NK cells, versus polyclonally expanded CD56^low^ NK cells (*p* < 0.04) (Figure 1 and Table 2). In addition, clonal CD56^low^ NK cells showed a pattern of expression of KIR molecules different from that of polyclonal CD56^low^ NK cells; accordingly, significantly lower percentages of CD158a^+^ and CD158b^+^cells, together with a higher intensity of expression of CD158e were found in the former group (Figure 1). In more detail, CD56^low^ NK cells from most monoclonal cases did not express any of the KIR molecules investigated (≤7% CD56^low^ NK cells were found to be positive for CD158a/b/e in 6/9 monoclonal *vs*. 0/12 polyclonal cases; *p* < 0.05), while CD161 expression was usually restricted either to virtually all or ≤ 5% clonal cells (Table 2). In contrast, patients with a polyclonal expansion of CD56^low^ NK cells usually showed predominant expression of one KIR (>50% of CD56^low^ NK cells) molecule in 6/11 polyclonal cases *vs*. 1/9 monoclonal cases (Table 2 and Supplementary Figure S1). Clonal CD56^low^ NK cells also showed significantly lower levels of granzyme B (mean fluorescence intensity -MFI- of granzyme B of 160 *vs*. 98 for cases with expanded polyclonal vs. clonal NK cells; *p* = 0.03). In turn, whereas two cases having clonal CD56^low^ NK cells were CD2^−^, clonal cases showed overall significantly higher levels of CD2 and lower amounts of CD38/cell *vs*. both normal and expanded polyclonal NK cells (*p* ≤ 0.002); finally, CD11c levels were greater in clonal *vs*. polyclonally expanded CD56^low^ NK cells (*p* = 0.01) (Figure 1).

**Table 2:** Expression of the CD158a/b/e killer-immunoglobulin-like receptors (KIR) and the CD94 and CD161 C-type lectin-like receptors on peripheral blood CD56^low^ NK cells from patients with clonal (*n* = 9) and polyclonal (*n* = 12*) expansions of CD56^low^ NK cells

	Percentage of CD56^low^ NK cells expressing each marker				
	CD158a	CD158b	CD158e	CD94	CD161
Clonal#1	20	58	6	100	40
Clonal#2	8	10	8	100	100
Clonal#3	39	20	46	100	87
Clonal#4	0	1	1	100	0
Clonal#5	0	1	1	100	5
Clonal#6	0	3	2	100	0
Clonal#7	1	2	2	100	2
Clonal#8	0	0	0	100	0
Clonal#9	0	7	0	100	100
**Median**	**0**	**3**	**2**	**100**	**5**
**Interquartile range**	**0–14**	**1–15**	**0.5–7**	**100–100**	**0–93**
**Minimum-maximum values**	**0–39**	**0–58**	**0–46**	**100–100**	**0–100**
Polyclonal#1	73	23	9	100	57
Polyclonal#2	10	10	0	81	35
Polyclonal#5	14	n.a.	9	47	32
Polyclonal#6	27	28	20	42	25
Polyclonal#7	28	15	11	40	78
Polyclonal#8	46	37	26	34	75
Polyclonal#9	3	43	0	56	n.a.
Polyclonal#10	75	15	6	65	61
Polyclonal#11	58	73	0	75	62
Polyclonal#12	6	76	0	80	20
Polyclonal#13	3	60	18	76	77
Polyclonal#14	29	70	0	72	37
**Median**	**27.5**	**37**	**7.5**	**68.5**	**57**
**Interquartile range**	**7–55**	**15–70**	**0–16.3**	**43.3–79**	**32–75**
**Minimum-maximum values**	**3–75**	**23–76**	**0–26**	**34–100**	**25–78**
***p*-values**	**0.007**	**0.003**	n.s.	**<0.0001**	n.s.

n.a.: not available; n.s.: Not statistically significant differences (*p* > 0.05) between clonal and polyclonal cases.

* In two out of the 14 cases with expanded polyclonal CD56^low^ NK cells, data about more than one killer receptor was not available, and therefore, these two cases were excluded from Table 2.

In contrast, CD56^low^ NK cells from all three groups of subjects displayed stable and similar reactivity for CD8, CD45RA and CD45 (Figure 1), in the absence of CD3, CD25, CD27, CD28 and CD197 (CCR7).

### Identification of the most discriminating immunophenotypic markers for the classification of CD56^low^ NK cells

**Figure 2:**
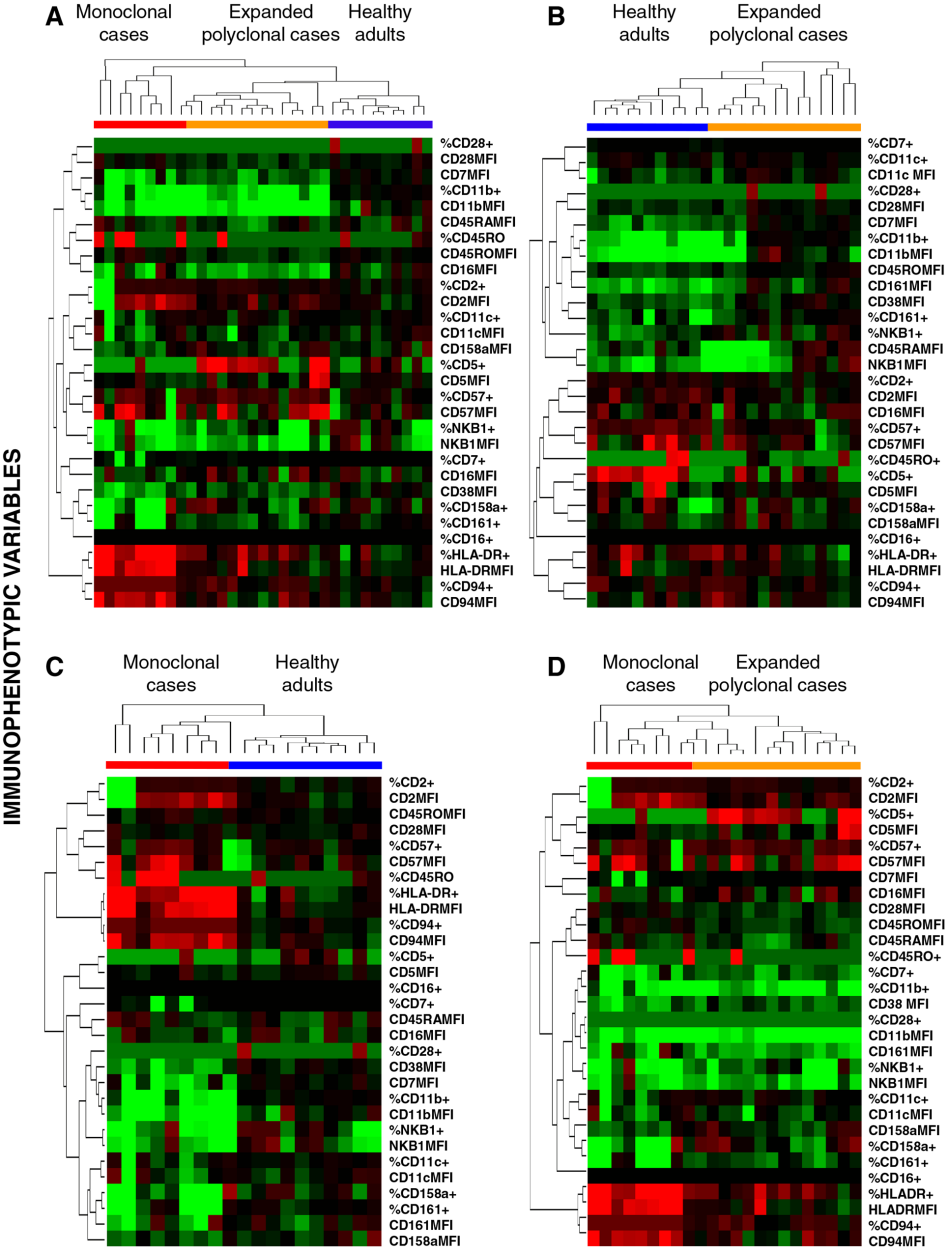
Unsupervised hierarchical clustering analysis of the immunophenotypic pattern of expanded monoclonal, expanded polyclonal and normal adult peripheral blood (PB) CD56^low^ NK cells Trees were generated based on the expression patterns observed on PB CD56^low^ NK cells for the 30 immunophenotypic variables included in the study, generated using the *Cluster and TreeView* software programs. **Panel A.** shows the clustering trees of the three groups included in the study, while **Panels B, C.** and **D.** show the trees generated for the following analyses: healthy adults *vs*. expanded polyclonal NK-cell cases (B); expanded monoclonal NK-cell cases *vs*. healthy adults (C) and; expanded monoclonal *vs*. expanded polyclonal patient groups (D), respectively. For each given marker, immunophenotypic data is expressed both as percentage of positive cells and as mean fluorescence intensity of positive cells –arbitrary relative linear units scaled from 0 to 10^4^cells–, except for CD38 and CD45RA, which are expressed only as MFI values (for a total of 30 immunophenotypic variables). Columns show the expression levels for a single sample; rows represent the relative levels of expression for a single immunophenotypic parameter, centered at the geometric mean of the expression levels observed for all samples. Red and green colors indicate values higher or lower than the mean, respectively; color intensity represents the magnitude of the deviation from the mean; black color represents no significant change in the expression levels for that sample *vs*. the mean.

Unsupervised hierarchical clustering analysis based on 30 immunophenotypic parameters confirmed the existence of three different, but partially overlapping groups of CD56^low^ NK cells, such unique phenotypic profiles being associated with expanded clonal and polyclonal NK cells, plus normal PB NK cells, respectively (Figure 2). The most relevant parameters for the discrimination between monoclonal NK cells from both control (normal) and expanded polyclonal NK cells included three overexpressed markers (HLADR, CD94 and CD45RO) and four down-regulated protein-associated variables (% CD158a^+^, % CD161^+^, % CD11b^+^ and CD38 MFI) (Figure 2) among the former group.

**Figure 3:**
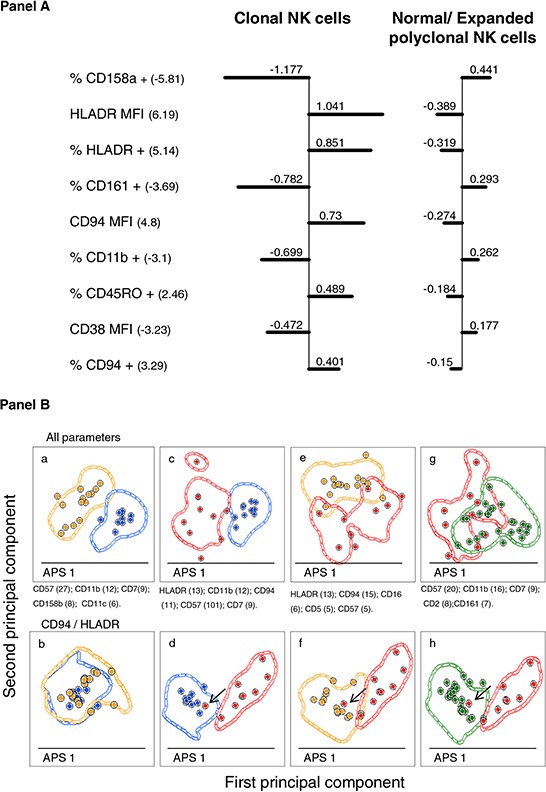
Identification of the most relevant phenotypic markers contributing to the discrimination between normal/polyclonal and monoclonal peripheral blood CD56^low^ NK cells **Panel A.** illustrates the **“Nearest-shrunken centroid” analysis**, showing the shrunken differences for the 7 immunophenotypic markers having at least 1 non-zero difference, selected as significant by SAM analysis. Left-sided and right-sided bars indicate lower and higher expression in each group with respect to the overall centroid, respectively. Markers are ordered from the greatest to the smallest difference. Numbers in brackets indicate d-Values: positive *d*-Values denote over-expression on clonal CD56^low^ NK cells, while negative *d*-Values indicate under-expression on clonal CD56^low^ NK cells. **Panel B.** shows pairwise PCA-based comparisons of the phenotypes of expanded monoclonal, expanded polyclonal and normal adult peripheral blood CD56^low^ NK cells. APS -Automated Population Separator function included in the INFINICYT software, corresponding to the principal components 1 (X axis) and 2 (y axis)- views showing the discrimination between CD56^low^ NK cells from: healthy adults (blue circles) *vs*. reactive polyclonal (orange circles) cases (a and b); healthy adults (blue circles) *vs*. clonal (red circles) cases (c and d); clonal (red circles) *vs*. polyclonal (orange circles) (e and f); and clonal (red circles) *vs*. both healthy adults and reactive (green circles) cases (g and h). Each circle represents one single case (median expression observed for all the phenotypic parameters evaluated). Contour lines in these plots represent one standard deviation curves. Panels a, c, e and g show mean principal component 1 *vs*. principal component 2 values for each case, considering all phenotypic parameters analyzed; the first four parameters contributing to the best discrimination between the two groups (ordered according to their percentage of contribution) are displayed at the bottom of each plot. Panels b, d, f and h show mean principal component 1 *vs*. principal component 2 values for each case, but considering only the CD94 and HLADR parameters. The arrow denotes the only misclassified case.

A predictive model (PM) based on the above referred discriminating parameters was then built and evaluated by applying it to the analysis of the 33 samples of the training set (10 healthy adult donors *plus* 9 monoclonal female cases *plus* 14 polyclonal female cases; Figure 3, Panel A). Based on this model, all but two samples (31/33; 94%), corresponding to CD94^low^/HLADR^−/low^ clonal CD56^low^ NK cells, were correctly classified, with a probability of correct classification of 81% for clonal cases and 98% for polyclonal/normal CD56^low^ NK-cell samples. Subsequently, the same PM was used to classify the remaining 37 patient samples with expansions of CD56^low^ NK cells, in which the HUMARA assay could not been performed and/or was not informative for the assessment of clonality. From these 37 cases, 11 were classified as being clonal and 26 as polyclonal/normal, with a probability of being correctly classified of 90% and 91%, respectively.

To further confirm the value of the above described phenotypic markers for the discrimination between monoclonal *vs*. polyclonal CD56^low^ NK cells, the PCA (principal component analysis) function of the INFINICYT software, was used. Despite clonal cases could not be clustered in a single group by PCA, they clearly separated from both normal and expanded polyclonal cases when phenotypic parameters were considered in the analysis (Figure 3, Panel Bg); such discrimination was even more clear on PCA when only the two most informative markers (HLADR and CD94) were used (Figure 3, Panel Bh). Actually, only one clonal case did not cluster separately from the group with expanded polyclonal CD56^low^ NK cells, due to the fact that NK cells from this discordant case had low CD94 expression levels, in the absence of HLADR. The INFINICYT reference data set generated upon excluding this case, was then used to classify the remaining 37 patient samples containing CD56^low^ NK cells of undetermined/unknown clonality (Supplementary Figure S2); then, each tested case was evaluated by the “fixed” PCA function and assigned to its nearest reference entity in the principal component (PC)1 *vs*. PC2 (APS plot in the INFINICYT software) space. Based on this approach, 11 cases were classified as monoclonal and 26 as normal/polyclonal cases, with a 100% concordance between the PCA-based and the PM-based classifications.

### Follow-up of CLPD-NK cases predicted to be clonal vs. polyclonal on immunophenotypic grounds

Clinical and hematological data were collected after long-term follow-up (median of 34 months, ranging from 3 to 156 months) in a subset of 21/37 CLPD-NK cases in which both the HUMARA assay and the analysis of somatic mutations of *STAT3/STAT5b* were not performed and/or were not informative (9 classified as clonal and 12 as polyclonal by the predictive models). Interestingly, in all 9 cases with undetermined clonality who were predicted to have clonal NK-cells, the absolute lymphocyte count remained over the normal range after a median follow-up of 29 months; even more, in 2/9 cases the lymphocyte count increased (from 4.3 to 10.2 × 10^9^/l and from 4.8 to 8.4 × 10^9^/l). Of note, one of these later patients died 4 years after first diagnosis, although the actual cause of death was unknown. In contrast, in 58% (7/12) of cases predicted to be polyclonal by the statistical predictive models described below, the lymphocyte count decreased and returned to normal values after a median follow-up of 31 months, while it remained stable and high in the other 5 cases. Most of these "polyclonal" CLPD-NK patients (*n* = 10/12) had no clinical symptoms at follow-up; the two symptomatic cases corresponded to two patients diagnosed with cancer who died due to their tumors, after NK-cell counts had decreased toward normal values.

## DISCUSSION

CLPD-NK has been recognized as a new provisional diagnostic entity in the WHO2008 classification of hematopoietic tumors, these cases being clearly different from aggressive mature NK-cell neoplasms such as extranodal NK/T cell lymphoma, nasal type and aggressive NK cell leukemia (ANKL) [11, 30, 31]. CLPD-NK usually shows an indolent clinical behavior; however, far from being a homogeneous group of diseases, CLPD-NK encompasses truly leukemic cases, some of which even show transformation to ANKL [32, 33], and reactive conditions, which might likely represent the extreme pole of an immunologic response to unknown stimuli [10]. In contrast to chronic expansions of clonal *vs*. reactive B and T cells, which can be more easily distinguished based on the analysis of the immunoglobulin/T-cell receptor gene rearrangements, CLPD of clonal *vs*. polyclonal NK cells are rather difficult to diagnose, among other reasons, because of the lack of a universal and specific marker for NK- cell clonality. This also concerns detection of *STAT3* somatic mutations which have recently been identified as a recurrent genetic abnormality in patients with CLPD-NK, since they are only present in between 18% and 30% of cases by conventional sequencing techniques [27, 34], in line with our findings. The challenge that assessment of clonality represents in CLPD-NK, together with the rarity of NK-cell tumors, undoubtedly have hampered our ability to standardize the diagnostic procedures for this entity, and hence, the clinical management of CLPD-NK patients.

At present, it is well-established that in most hematological malignancies (e.g. acute leukemias, B- and T-cell CLPD and plasma cell disorders) neoplastic cells from virtually every patient display aberrant phenotypes, that can be used for the distinction of such tumor cells from their normal counterparts [35, 36,]. Of note, such aberrant phenotypes are not exclusive of malignant leukemias and lymphomas, as they can be also found at similar frequencies in benign conditions, e.g. monoclonal B-cell lymphocytosis (MBL) [37] and monoclonal gammapathy of undetermined significance (MGUS) [38]. Consequently, the "leukemia-associated phenotypes" can also be considered to represent markers for clonality. Based on this hypothesis, here we investigated for the first time the utility of a broad panel of immunophenotypic markers assessed by flow cytometry, as surrogate markers for the classification of CLPD of CD56^low^ NK cells into clonal *vs*. polyclonal cases. For this purpose, we comparatively analyzed the immunophenotypic profile of PB CD56^low^ NK cells from female patients in whom the clonal *vs*. polyclonal nature of the expanded cells was previously known (e.g. by the HUMARA X-chromosome inactivation assay performed on highly purified cells) to that of normal adult PB CD56^low^ NK cells. Phenotypic analyses included a large panel of NK-cell-associated markers, consisting of signaling (e.g. CD2, CD5, CD7, CD8) and adhesion molecules (e.g CD11b and CD11c), activation-related markers (e.g. CD38, CD45RO and HLADR), IL2 receptors (e.g. CD25 and CD122), maturation-associated proteins (e.g. CD57), cytotoxic enzymes (e.g. granzyme B and perforin) and receptors involved in the recognition of target cells (e.g. CD16, the CD158a, CD158b and CD158e natural-killer immunoglobulin-like receptors –KIR- and the CD94 and CD161 C-type lectin-like receptors), among other molecules. Based on the above referred markers, expanded CD56^low^ NK cells from both monoclonal and polyclonal patients showed an overlapping immunophenotypic profile consistent with that of late-stage activated NK cells [5], which was clearly different from the one displayed by normal CD56^low^ NK cells. These findings support the hypothesis that both clonal and polyclonally expanded NK cells could emerge as a consequence of chronic antigen stimuli. In this regard, CD56^low^ NK cells from CLPD patients showed higher expression of CD57, granzyme B and perforin than normal control cells, together with a CD2^high^/CD7^low^ phenotypic pattern, upregulation of CD94 and HLADR and lower expression of CD11b, CD38 and CD161; while the former marker profile is typical of late-maturation stage cells, the later is usually featured on activated NK cells [5].

Recent studies have evaluated the expression profile of natural cytotoxicity receptors [24] and different killer cell immunoglobulin-like receptors [21, 31], showing a skewed repertoire of KIR receptors on the expanded cells from CLPD-NK patients [24, 29]; in turn, others have reported bright CD94 expression on pathological cells from CLPD-NK patients [24, 29]. However, despite the biological findings and the interest of these reports, in none of them the monoclonal *vs*. polyclonal nature of the expanded NK cells was investigated, in contrast to what was achieved here. Thus, the potential contribution of immunophenotyping to the differential diagnosis between expanded clonal and polyclonal CD56^low^ NK cells in CLPD-NK, would be of utmost clinical relevance, specially if we take into account that there were no significant clinical differences between the two patient groups, in line with previous observations [7, 8, 40]. In this regard, and despite the similarities between the expanded clonal and polyclonal NK cells, detailed analysis of the pattern of expression of individual markers already highlighted significant differences between the two patient groups, including a greater expression of CD94 and HLADR (both in terms of percentage of cells and expression levels/cell of both markers), as well as CD2, together with lower positivity for the CD158a/b/e KIR molecules, CD38 and granzyme B in expanded clonal *vs*. polyclonal CD56^low^ NK cells. Of note, expanded CD56^low^ NK cells from most monoclonal cases lacked on expression of all KIR investigated, in contrast to normal/polyclonal NK cells from both reactive cases and healthy controls; these findings support and extend on previous studies that reported a skewed KIR repertoire for LGL-NK cell leukemias vs. normal NK cells [24]; in addition, here we show for the first time that most expanded CD56^low^ NK cells from individual reactive/polyclonal cases usually expressed one single (exceptionally two) KIR molecules, a feature that others have considered as a surrogate marker for clonality [24]. Further studies including a higher number of KIR and other killer receptor molecules analyzed on larger series of patients with CD56^low^ NK-cell expansions vs. healthy donors would be required, to definitively clarify this apparent discrepancy.

In order to identify a more robust NK-cell phenotypic profile that could be prospectively applied to the diagnosis of clonality in CLPD-NK patients, multivariate analyses were performed. As a result, unsupervised hierarchical clustering analysis showed that the most relevant parameters contributing to the distinction between monoclonal *vs*. both expanded polyclonal and normal CD56^low^ NK cells included overexpression of HLADR and CD94, together with CD45RO, and downregulation of CD158a, CD38, CD11b and CD161. Based on these markers, cases were segregated into three groups, polyclonal/reactive CLPD-NK cases being located between the normal and the monoclonal NK-cell groups. Based on these results, a predictive model was constructed using the above markers and the training set of cases; such model provided to properly classify all but two clonal CLPD-NK cases, whose CD56^low^ NK cells did not express high levels of HLADR and CD94. These observations also indicated that the phenotypic profile that would mostly contribute to the diagnosis of clonality of CLPD-NK strongly relies on the existence of a strong and more homogeneous expression of both HLADR and CD94, in such a way that when clonal NK cells show a different HLADR/CD94 profile, a high overlap with the phenotype of polyclonal (particularly reactive) NK cells might exist. This was further confirmed by PCA, which showed that the two most useful phenotypic parameters for the identification of clonal *vs*. normal/polyclonal CD56^low^ NK cells were HLADR and CD94. Actually, only one clonal case did not cluster separately from the expanded polyclonal CD56^low^ NK-cell group in the PCA, such a case showing a CD94^low^/HLADR^low^ immunophenotypic profile.

More interestingly, when the two predictive models were applied for the prospective classification of the 37 CLPD-NK cases in which the HUMARA assay could not be performed and/or it was not informative, they showed a 100% concordance for the classification of this group of cases into the monoclonal (*n* = 11) and the polyclonal (*n* = 26) NK-cell categories. Follow-up of most of these patients was highly concordant with the predicted monoclonal vs. polyclonal nature of the expanded PB CD56^low^ NK cells at diagnosis. Thus, in two patients predicted to have clonal NK cells the absolute number of lymphocytes was increased with time, while in all other cases from this group kept stable over the normal range; in contrast, the lymphocyte count returned to normal values in near two thirds of cases predicted to be polyclonal and remained stable in the other third.

Altogether, these results provide the basis for prospective usage of altered NK-cell immunophenotypes, in the absence of other universal markers for NK-cell clonality, as a surrogate marker for the distinction between clonal and reactive NK-cell expansions in the diagnostic work-up of CLPD-NK. The most discriminating phenotypic profile that contributed to such distinction consist of a strong and more homogeneous expression of CD94 and HLADR and, to a lesser extent also, downregulation of CD158a, CD38, CD11b and CD161.

## MATERIALs AND METHODS

### Patients and samples

A total of 60 patients who showed increased numbers of circulating PB CD56^low^ NK cells, referred to the Cytometry Service of the University of Salamanca (NUCLEUS) during a 12-year period of time, were studied. Median age of the patients −29 males and 31 females- was of 65 years (range: 25 to 86 years). Inclusion criteria were: presence of increased numbers of PB CD56^low^ NK cells (>2 × 10^9^/L and/or > 20% NK cells from all circulating lymphocytes), for more than 6 months, or identification of phenotypically aberrant CD56^low^ NK cells, regardless of their actual numbers. An additional group of 10 adult healthy volunteers (3 males and 7 females; median age of 47 years, ranging from 27 to 83 years) with normal blood cell counts, in the absence of any apparent or known disease was also included in the study.

PB samples from both the patients and the controls were collected into tubes containing K3-EDTA, after written informed consent was given by each subject, according to the Declaration of Helsinki. The study was approved by the Ethics Committee of the University Hospital of Salamanca (Salamanca, Spain).

### Flow cytometry immunophenotypic studies

Enumeration of PB lymphoid cell subsets, including CD56^low^ NK cells, was performed using the *Lymphoclonal^TM^ reagent* (Cytognos SL, Salamanca, Spain), which contains a mixture of seven antibodies (Ab) [fluorescein isothiocyanate (FITC)/phycoerythrin (PE)/peridin chlorophyll protein-cyanin 5.5 (PerCP-Cy5.5)/allophycocyanin (APC)]: anti-human immunoglobulin light chain λ (Igλ) + CD8/anti-human Igκ + CD56/CD19 + CD4/CD3. Further immunophenotypic characterization of PB CD56^low^ NK cells was performed using the following 4-color combinations of fluorochrome-conjugated -FITC/PE/PE-cyanin 5 (PE-Cy5) or PerCP-Cy5.5/APC- monoclonal Ab (MAb): CD7/CD5/CD56/CD3; CD2/CD28/CD56/CD3; CD57/CD11c/CD56/CD3; CD38/CD11b/CD56/CD3; CD94/HLA-DR/CD56/CD3; CD45RA/CD197/CD56/CD3; CD27/CD45RO/CD56/CD3; CD16/CD158e (NKB1)/CD56/CD3; CLA/CD158b (NKAT2)/CD56/CD3; CD158a (NKAT1)/CD161/CD56/CD3; CD122/CD25/CD56/CD3 and cytoplasmic (Cy) perforin/Cy granzyme B/CD56/CD3. The sources and specificities of the MAb reagents used have been previously described in detail [26, 28]. Cell staining was performed on whole blood samples, using a well-established stain-and-then-lyse method, as previously reported [26, 28]. Combined staining for surface antigens and intracellular molecules was performed using the *Fix & Perm*^TM^ reagent kit (Invitrogen, Carlsbad, CA, USA), according to the recommendations of the manufacturer. Data acquisition was performed immediately after completion of sample preparation, in a FACSCalibur flow cytometer -Becton Dickinson Biosciences (BD), San Jose, CA, USA- using the CellQUEST^TM^ software program (BD). For data analysis, the INFINICYT^TM^ software program (Cytognos), was used. In each case, CD56^low^ NK cells were defined as those CD56^low^/CD3^−^ events included in a broad forward (FSC) *vs*. sideward light scatter (SSC) lymphocyte region. For each antigen analyzed, both the percentage of positive CD56^low^ NK cells and its intensity of expression -reflected by the MFI expressed in arbitrary relative linear units scaled from 0 to 10^4^-, were evaluated.

### HUMARA assay

In order to confirm the monoclonal *vs*. polyclonal nature of the expanded/aberrant CD56^low^ NK cells, the pattern of inactivation of the human androgen receptor coded in chromosome X was studied in 25/31 female patients. The methylation status of the human androgen receptor gene was evaluated with a HUMARA polymerase chain reaction (PCR)-based assay [25, 41, 42, 43] for paired fractions of purified CD56^low^ NK cells (mean purity of 98% ± 1.2%; range: 94% to 99%) and the corresponding NK-cell depleted leukocyte fraction from the same subject (*n* = 25). NK cells were purified from mononuclear PB cells (obtained after Ficoll-Paque^TM^ density gradient centrifugation) by a single immunomagnetic depletion step using the *Human NK-Cell Enrichment Kit* (StemStep^TM^, StemCell Technologies Inc, Vancouver, BC, Canada) and an AutoMACS magnetic cell separator (Miltenyi Biotec, Bergisch Gladbach, Germany), strictly following the recommendations of the manufacturer. Purified populations were used to extract genomic DNA (gDNA) using the QIAamp mini- or micro-DNA extraction Kits (QIAGEN, Valencia, CA). Digestion of genomic DNA was performed with the HapII methylation-sensitive restriction endonuclease and subsequent PCR amplification of the methylated (inactivated) alleles, as previously described [25, 41, 42]. According to previous reports, a case was considered to be monoclonal when the corrected allele ratio was of ≤0.33 or ≥3, indicating that one of the parental alleles was represented at an excess of ≥50% *vs*. the other allele [43].

### Analysis of *STAT3* and *STAT5b* somatic mutations

*STAT3* and *STAT5b* gene mutations were analyzed on gDNA extracted from highly purified CD56^low^ NK cells -according to the techniques described above- from a total of 9 cases which had also been evaluated by the HUMARA assay, from which 4 corresponded to monoclonal and 5 to polyclonal cases. Previously reported PCR primers were used to amplify known somatic mutational hotspot regions in the *STAT3* and *STAT5b* genes, located at *STAT3* SH2 exons 19–20 and 21, as well as at *STAT5b* SH2 exon 16 [27, 34, 44]. The amplified products were sequenced by conventional techniques at the *Genomic Unit* of the *Cancer Research Center* (IBMCC, USAL-CSIC, Salamanca, Spain); all mutations were screened by bidirectional sequencing and scored as pathogenic mutations on the basis of the observation that they were not detected in paired cell subsets other than NK cells. The sequences were analyzed using the Chromas Lite Sequencing Software (South Brisbane, Australia).

### Statistical methods

Conventional statistics –nonparametric Kruskal-Wallis and Mann-Whitney U tests for continuous variables, or the Pearson's χ2 and Fisher exact tests for categorical variables– were performed using the SPSS software program (IBM SPSS Statistics, IBM, Armonk, NY, USA). P values ≤ 0.05 were considered to be associated with statistical significance.

Multivariate analysis of markers differentially expressed in normal and reactive *vs*. clonal CD56^low^ NK cells was performed in four consecutive steps. Only healthy subjects (*n* = 10) and those female patients found to be heterozygous for the HUMARA assay (*n* = 23 female cases tested; 9 monoclonal and 14 polyclonal) were included in the analysis. Prior to the analysis, data about the immunophenotype of CD56^low^ NK cells – percentage of cells positive for each individual marker, as well as the MFI values of CD56^low^ NK cells positive for each marker (*n* = 30 variables)– was normalized using the mean value obtained for each individual variable in normal PB NK cells and logarithmically (base 2) transformed. Afterward (step 1), both unsupervised and supervised hierarchical clustering analyses based on the average-linkage method and an Euclidean metric [45] were applied (Cluster 3.0 and Tree View software, Stanford University, Stanford, CA, USA) [46]. In a second step, a Significant Analysis of Microarrays (SAM) algorithm was built -with the same 30 variables used in step 1- in order to identify those markers showing a statistically significantly different expression in the clonal (*n* = 9) *vs*. polyclonal classes (*n* = 14 cases plus 10 healthy controls). Data was permutated over 100 cycles by using the two-class (unpaired) format and the statistically relevant variables (*T*-test) were selected, based on the lowest false discovery ratio (FDR < 2.2) and the *q*-value (0) [47] obtained. In the following step (third step), those variables identified to be significantly associated with different classes were used to build a class predictor (weighted voting algorithm with a leave-one-out cross validation), that included the smallest combination of variables for correct classification of CD56^low^ NK-cell expansions as monoclonal *vs*. polyclonal. A training set containing all above referred 33 cases (10 healthy adult donors *plus* 9 monoclonal female cases *plus* 14 polyclonal female cases) was used to train the classifier, and the accuracy of the predictor was subsequently tested in a set including all the 60 patients studied and who presented with abnormal NK-cell numbers and/or phenotypes, using the nearest shrunken centroids method (PAM software; University of Stanford, Stanford, CA, USA) [48]. In the last (fourth) step, principal component analysis (PCA) was applied to flow cytometric data from individual cells, in order to confirm the value of the individual variables selected for correct classification of monoclonal *vs*. polyclonal nature of a given expansion of CD56^low^ NK cells. For this purpose, a reference data set was built with data from healthy controls (*n* = 10) and those patients proved to have polyclonal (*n* = 14) and monoclonal (*n* = 9) NK-cell expansions by the HUMARA assay, using the merge and calculation functions of the INFINICYT^TM^ software, as previously described [49]. Unsupervised PCA was then performed for the reference database and data was then visualized in a 2-dimensional plot defined by the first (PC1) *vs.* second (PC2) principal component (APS plot in the INFINICYT software) [50, 51] for the distinction among different clusters of NK cells.

## SUPPLEMENTARY FIGURES


