# Supplementary material for: Phenotypic profile of expanded NK cells in chronic lymphoproliferative disorders: a surrogate marker for NK-cell clonality
Source: Oncotarget. 2015 Nov 6;6(40):42938–51. doi: 10.18632/oncotarget.5480 (PMC4767482; doi:10.18632/oncotarget.5480)
Supplement: Supplementary file 1 [file oncotarget-06-42938-s001.pdf]

SUPPLEMENTARY FIGURES

Percentage of CD56[low] NK cells expressing each marker

	CD158a	CD158b	CD158e	CD94	CD161
Clonal#1	20	58	6	100	40
Clonal#2	8	10	8	100	100
Clonal#3	39	20	46	100	87
Clonal#4	0	1	1	100	0
Clonal#5	0	1	1	100	5
Clonal#6	0	3	2	100	0
Clonal#7	1	2	2	100	2
Clonal#8	0	0	0	100	0
Clonal#9	0	7	0	100	100
Polyclonal#1	73	23	9	100	57
Polyclonal#2	10	10	0	81	35
Polyclonal#5	14	NA	9	47	32
Polyclonal#6	27	28	20	42	25
Polyclonal#7	28	15	11	40	78
Polyclonal#8	46	37	26	34	75
Polyclonal#9	3	43	0	56	NA
Polyclonal#10	75	15	6	65	61
Polyclonal#11	58	73	0	75	62
Polyclonal#12	6	76	0	80	20
Polyclonal#13	3	60	18	76	77
Polyclonal#14	29	70	0	72	37
NPB#1	12	35	32	52	80
NPB#2	25	28	31	30	94
NPB#3	31	37	17	41	83
NPB#4	43	41	4	70	41
NPB#5	11	21	19	45	56
NPB#6	20	45	29	72	74
NPB#7	9	32	0	48	84
NPB#8	37	32	0	27	96
NPB#9	38	28	8	41	89
NPB#10	43	44	31	40	92

% of positive cells
- 0%
- 1-5%
- >5 - 15%
- >15 - 30%
- >30 - 50%
- >50 - 75%
- >75%

% of positive cells
- 0%
- 1-5%
- >5 - 15%
- >15 - 30%
- >30 - 50%
- >50 - 75%
- >75%

% of positive cells
- 0%
- 1-5%
- >5 - 15%
- >15 - 30%
- >30 - 50%
- >50 - 75%
- >75%

NA: not available. NPB: normal peripheral blood (from healthy adult donors)

Supplementary Figure S1: Expression of the CD158a/b/e killer-immunoglobulin-like receptors (KIR) and the CD94 and CD161 C-type lectin-like receptors on peripheral blood CD56[low] NK cells from patients with clonal and polyclonal expansions of CD56[low] NK cells vs. normal subjects, analyzed in each individual case. Color codes have been arbitrarily selected according to the percentage of cells being positive for each individual marker, to better highlight the differences in the repertoire of killer-associated molecules among the three groups.

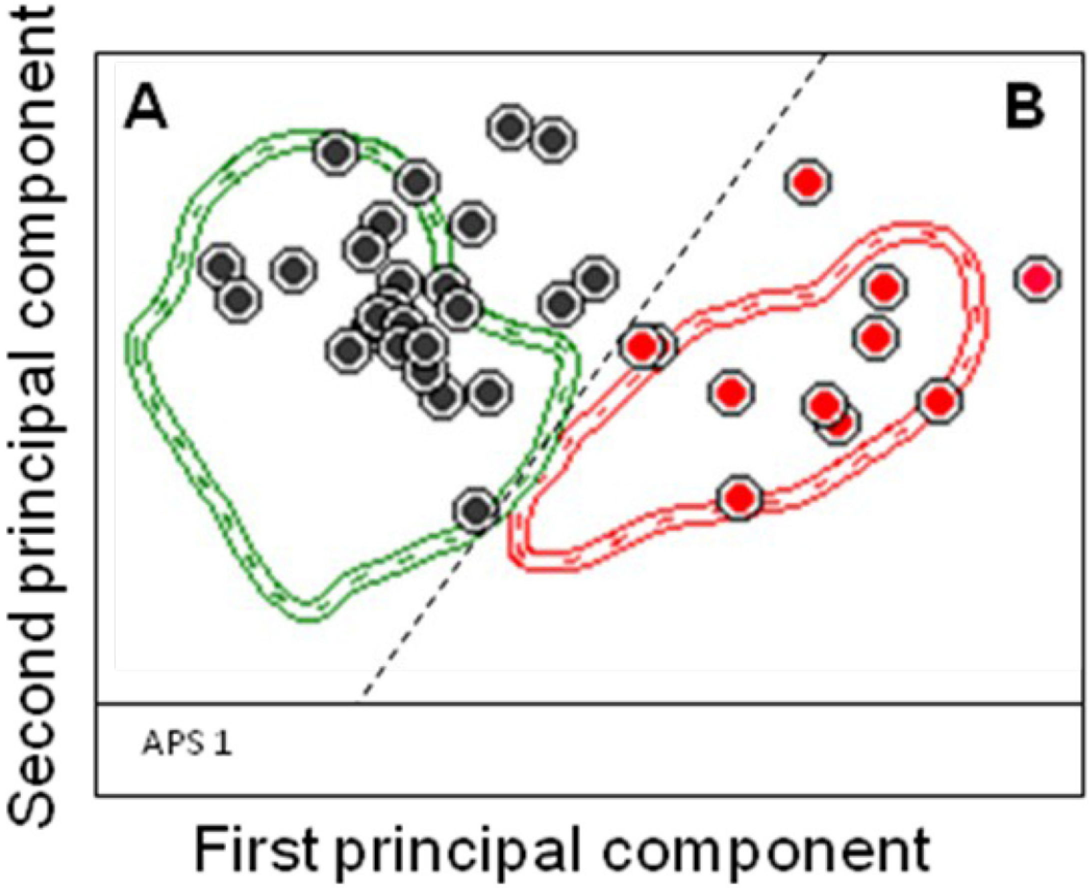

Supplementary Figure S2: PCA-based comparison (APS view of PC1 *vs.* PC2) of the whole immunophenotypic profile of CD56^{low} NK cells from those 37 cases in which clonality assessment could not be performed and/or was not informative, projected on the contour lines representing one standard deviation curves of CD56^{low} NK cells from both normal (healthy adults) and reactive/polyclonal (green contour line) vs. clonal (red contour line) cases from the test series. In the APS plot, the 37 cases used in the validation phase are represented as individual cases. Clonal and polyclonal cases used in the testing phase have been deleted from the plot, and only their contour (standard deviation) lines are shown; each circle represents one single case (median expression levels observed for all the phenotypic parameters evaluated in PC1 *vs*. PC2). **A.** Cases predicted as being polyclonal by the SAM predictive model (n = 26; black dots) and **B.** cases predicted as being clonal by the SAM predictive model (n = 11; red dots) fully overlapped with those predicted by the PCA displayed here.
